# Enhancing system-wide implementation of opioid prescribing guidelines in primary care: protocol for a stepped-wedge quality improvement project

**DOI:** 10.1186/s12913-018-3227-2

**Published:** 2018-06-05

**Authors:** Aleksandra E. Zgierska, Regina M. Vidaver, Paul Smith, Mary W. Ales, Kate Nisbet, Deanne Boss, Wen-Jan Tuan, David L. Hahn

**Affiliations:** 1 Department of Family Medicine and Community Health, Wisconsin Research and Education Network, School of Medicine and Public Health, University of Wisconsin-Madison, 1100 Delaplaine Court, Madison, WI 53715 USA; 2 Interstate Postgraduate Medical Association, P.O. Box 5474, Madison, WI 53705 USA

**Keywords:** Opioid analgesics, Chronic pain, Quality improvement, Healthcare systems, Healthcare quality, Access and evaluation

## Abstract

**Background:**

Systematic implementation of guidelines for opioid therapy management in chronic non-cancer pain can reduce opioid-related harms. However, implementation of guideline-recommended practices in routine care is subpar. The goal of this quality improvement (QI) project is to assess whether a clinic-tailored QI intervention improves the implementation of a health system-wide, guideline-driven policy on opioid prescribing in primary care. This manuscript describes the protocol for this QI project.

**Methods:**

A health system with 28 primary care clinics caring for approximately 294,000 primary care patients developed and implemented a guideline-driven policy on long-term opioid therapy in adults with opioid-treated chronic non-cancer pain (estimated *N* = 3980). The policy provided multiple recommendations, including the universal use of treatment agreements, urine drug testing, depression and opioid misuse risk screening, and standardized documentation of the chronic pain diagnosis and treatment plan. The project team drew upon existing guidelines, feedback from end-users, experts and health system leadership to develop a robust QI intervention, targeting clinic-level implementation of policy-directed practices. The resulting multi-pronged QI intervention included clinic-wide and individual clinician-level educational interventions. The QI intervention will augment the health system’s “routine rollout” method, consisting of a single educational presentation to clinicians in group settings and a separate presentation for staff. A stepped-wedge design will enable 9 primary care clinics to receive the intervention and assessment of within-clinic and between-clinic changes in adherence to the policy items measured by clinic-level electronic health record-based measures and process measures of the experience with the intervention.

**Discussion:**

Developing methods for a health system-tailored QI intervention required a multi-step process to incorporate end-user feedback and account for the needs of targeted clinic team members. Delivery of such tailored QI interventions has the potential to enhance uptake of opioid therapy management policies in primary care. Results from this study are anticipated to elucidate the relative value of such QI activities.

**Electronic supplementary material:**

The online version of this article (10.1186/s12913-018-3227-2) contains supplementary material, which is available to authorized users.

## Background

Chronic non-cancer pain (“chronic pain”) is common, affecting over 100 million Americans [1]. It is often refractory to existing treatments, and many patients are prescribed opioids to reduce pain and disability. However, long-term opioids are controversial for chronic pain and have been linked to dose-dependent harm, including addiction and overdose death [2, 3]. Prescribed opioids serve as the main drug supply for approximately 85% of those who misuse opioids [4]. In the US, opioid-related overdose deaths have dramatically increased, making this a national public health crisis.

Systematic implementation of guidelines for opioid therapy has the potential to reduce inappropriate prescribing and its harmful effects [5–8]. Primary care clinicians account for about half of opioid prescribing [9, 10], thus primary care clinical teams are a logical target for quality improvement (QI) initiatives focused on improving opioid prescribing practices. A modest reduction in opioid prescribing rates was noted in a single academic medical system after a month-long QI effort that focused on the dissemination of information on opioid prescribing guidelines at meetings and via individual in-person or email communication with primary care clinicians [7]. A QI project at two rural emergency departments in Maine aimed at reducing prescribing of controlled substances for painful dental conditions led to an absolute reduction in opioid prescribing by 17% ([8]. A multi-pronged, statewide effort in Utah, consisting of formal presentations and ongoing QI efforts with primary care physicians, led to a 14% decrease in the state’s opioid-related deaths [5].

Dissemination of evidence-based recommendations into routine practice is critical for system-wide QI. Historically, however, adoption of guidelines has been slow and challenging [11], and research on effective methods for dissemination and implementation of guidelines is limited [12, 13]. In addition, guidelines on opioid therapy management are complex and based largely on expert consensus with limited research evidence, factors that likely affect the adoption of these guidelines in routine care [10, 14–16].

The project team therefore decided to develop, execute, and evaluate the impact of a tailored, multi-pronged QI intervention aimed at increasing primary care clinicians’ adherence to guideline-recommended practices for opioid therapy in chronic pain. Coincidentally, the local health system was initiating a guideline-driven opioid management policy for this patient population. The routine rollout efforts by the health system to implement this policy served as a platform on which to build and test the effects of a tailored, enhanced QI intervention, targeting safe and competent opioid prescribing. This report describes the design, development, and methods for execution of the QI intervention. The aim of this project is to test if an enhanced QI intervention can improve implementation of guideline-recommended opioid prescribing practices in primary care, compared to the routine rollout efforts of the health system. Future reports will describe the outcomes of this QI project.

## Methods/design

### Project aim

The studied health system had planned to implement a policy for the management of long-term opioid therapy in adults with chronic non-cancer pain (“opioid policy”) in its primary care family medicine (FM) and general internal medicine (GIM) clinics. The project team, comprised of physicians, researchers and educators, an electronic health record (EHR) database analyst, and a biostatistician, hypothesized the health system’s planned routine rollout approach to the implementation of opioid management policy may be suboptimal due to the complexity of opioid prescribing guidelines, the variety of team cultures and practices within the system, and the expected discomfort of clinicians in relation to the topic and complexity of the target patient population [14–19]. The project team proposed and developed a multi-pronged QI intervention aimed to augment the health system’s routine rollout implementation efforts, and designed an outcome evaluation plan to rigorously test the intervention effectiveness. Institutional Review Board review was not required because, in accordance with federal regulations, this was deemed a QI project not constituting research, as defined under 45 CFR 46.102(d).

### Settings/target population

#### Target population

In January 2016, the health system provided care for 293,927 primary care patients, including 204,680 adults, defined as 18 years old or older, across its 28 primary care clinics (18 FM; 10 GIM). Among adult patients, 3980 (1.9%) were estimated to be treated with opioids for at least 3 months for chronic pain (“target population”). Among target population patients (59% women; mean age: 53.3 ± 14.2 years), 38.8% were prescribed opioids at ≥50, and 26.0% at ≥90 morphine milligram equivalent (MME) per day. According to the Centers for Disease Control and Prevention guidelines, the 50 MME/day threshold is a recommended maximum dose for most opioids, with doses at or above 90 MME/day recommended to be avoided [16]. In the target population, 39% were co-prescribed benzodiazepines and opioids (based on the “active medication” list), 64.7% had a documented treatment agreement, 32.8% completed urine drug testing, and 21.9% completed a depression screening using a validated screening tool in the prior 12 months.

### Health System’s opioid policy and implementation efforts (“routine rollout”)

The health system’s opioid policy was finalized in June of 2015 and based on existing guidelines [14, 15]. The policy was developed by a multidisciplinary panel of clinicians, pharmacists, scientists, and policy-implementation specialists. It was designed to target adult primary care patients who were treated with long-term opioids for chronic non-cancer pain. The policy excluded those under hospice care, with life expectancy shorter than 6 months, or cancer pain. The health system’s Information Technology team developed the interface and tools in the EHR to be compatible with, and facilitate the implementation of, the opioid policy by primary care clinical staff. The policy recommendations included the initiation and regular update of treatment agreements; urine drug testing; screening for depression and the risk of opioid misuse; checking the state’s Prescription Drug Monitoring Program (PDMP) database; and documentation in the EHR of the chronic pain diagnosis, clinical progress, and treatment plan (Table 1).

**Table 1:** Outline of the recommendations of the health system’s opioid management policy

Item	Summary of the Policy-recommended Components
Recommended components of opioid therapy management	
Problem List	1. Document diagnosis of chronic pain and source of pain
	2. Document information related to relevant prescribed medications:
	a. Details of opioid prescription, with allowed quantity per given time period b. Name and location of designated pharmacy c. Date when treatment agreement was most recently signed d. Urine drug testing findings
	3. PDMP review: date of last review, finding summary, e.g., consistent or inconsistent with prescription record
	4. Document care plan
	5. Add comments helpful to other providers, e.g., those covering in your absence
	6. Update at least annually and when any changes occur
Care Plan Components	1. Treatment goals: pain severity (BPI), function (BPI/other)
	2. Treatment plan (medications, exercise, physical or occupational therapy, mental health related therapies, CAM therapies, specialty consults)
	3. Contingency plan for care outside PCP office
	4. Update at least annually and when any changes to care plan
Treatment Agreement	1. Serves as informed consent to long-term opioid therapy
	2. Scan new or updated signed treatment agreement into the EHR
	3. Update treatment agreement annually and when any changes to care plan
	4. Deactivate treatment agreement after opioids are no longer prescribed
Urine Drug Testing	1. Complete urine drug testing annually or more frequently as needed
	2. Perform confirmatory testing for unexpected results of a screening test
	3. Document findings
Prescription Refills	1. Prescription for controlled substances should be filled at one agreed upon pharmacy, which is noted in the treatment agreement
	2. Prescriptions for Schedule II medications can be mailed to pharmacy only
	3. Patient may sign a release form to designate up to 2 appointees who can pick up prescriptions for Schedule II medications with photo ID
PDMP	1. Document findings of the PDMP database review at least annually.
Approach to treatment agreement violation	
Minor Infractions	1. Patient should be contacted by prescribing provider; discussion documented
	2. Reassess and update care plan and treatment agreement as needed
Major Infractions	Follow minor infraction steps above; in addition:
	1. If opioid therapy is discontinued, provide, when appropriate: a. opioid taper instructions and prescription(s) to accomplish the taper b. prescriptions for non-opioid medications for opioid withdrawal symptoms
	2. Document reason for the discontinuation of opioid therapy
	3. Deactivate treatment agreement when opioid treatment is completed
	4. Communicate with other treating clinicians
	5. Contact Patient Relations; discuss placing a flag, if needed, in medical record by the Department of Pharmacy
	6. Continue non-opioid treatment
	7. If all care is planned to be terminated, discuss “No further service” with Patient Relations
Suspected Misuse or Use Disorder	1. Consider referring to addiction medicine specialist
	2. If safe, continue modified or current opioid therapy until plan is in place with addiction specialist
	3. Consider following the steps as for major violation of the treatment agreement

*BPI* Brief Pain Inventory, *CAM* Complementary and Alternative Medicine, *EHR* Electronic Health Record, *PCP* Primary Care Provider, *PDMP* Prescription Drug Monitoring Program

The health system developed a policy implementation training program for its primary care prescribers and clinical support staff that was pilot-tested from September – November 2015 in one FM and two GIM community clinics. Based on feedback from the pilot sites, the health system refined the implementation methods and initiated the system-wide implementation effort (“routine rollout”) in February 2016. Project team members met four times with the health system leadership; observed the health system-led pilot implementation efforts in 3 clinics (3 in-person educational training sessions on policy implementation; 3 teleconference debriefing sessions on the pilot-clinic experiences); and attended all of the system-wide policy rollout activities. The “routine” system-wide rollout consisted of: 1) a single, in-person 1-h introductory meeting for groups of clinicians; 2) a 1-h online training module for staff to be completed under the clinic managers’ supervision; and 3) two follow-up teleconference sessions led by the health system’s clinical knowledge implementation team to address any questions or comments from prescribers and other clinical staff.

### Design

#### Overall design

Based on a sample size calculation, described in the Statistical Analysis section, the project team proposed to enroll 9 of the 28 health system’s primary care clinics into a stepped-wedge 18-month trial. The clinics with the highest rates of opioid prescribing for adult patients with chronic pain will be approached first. Each enrolled clinic will start as a control site; then, in waves of 3, clinics will sequentially receive the intervention until all become intervention sites. Use of a stepped-wedge design, coupled with outcome measures assessed via EHR-based data, will allow an efficient, rigorous and controlled evaluation of the effectiveness of the proposed intervention that, if proven successful, can be rapidly disseminated across an entire health system.

#### QI intervention

The QI intervention was developed over a 12 month period (January–December 2015) and designed to augment the “routine rollout” implementation efforts of the health system.

The intervention was informed by the following: 1) the goals of the health system’s new opioid management policy; 2) feedback from the health system’s leadership, the policy implementation team and participants at the pilot clinics; and 3) the project team’s combined expertise in primary care, addiction medicine, opioid therapy management, implementation science, health services research, including knowledge of the available EHR-based outcome measures and clinical “charting” tools, medical education design and implementation, practice facilitation and statistical analysis. The intervention educational content was updated as needed to reflect changes in relevant guidelines or law.

The intervention consists of several components (Table 2):

1. *Academic Detailing* At the beginning of each intervention, two physician study members (AZ or DH) deliver an on-site 1-h presentation to clinic staff about the study goals; summary of the health system’s opioid policy and dangers of co-prescribing opioids and benzodiazepines; and an overview of the QI intervention and available educational credits. The presentation includes 32 slides to be delivered over approximately 30 min, with the remaining time designated for discussion with the clinic staff.
2. *Online Educational Modules* The health system’s opioid policy, feedback from the pilot clinics, and the expertise of team members and invited external experts shaped the development of two online educational modules. Both modules incorporate evidence-based, system-specific, process-related information to make the knowledge gained relevant to “real-life” primary care in the health system’s clinics. Each module consists of 20–21 questions, delivered via email (1–2 questions every 1–2 days), with multiple-choice answers and a brief rationale for correct and incorrect answers. The “Responsible Opioid Prescribing” module emphasizes real-life implementation of the opioid management policy in the context of the health system-specific clinical settings. The “Shared Decision Making” module includes clinical cases linking information about shared decision-making principles to the care for patients with opioid-treated chronic pain.
3. *Practice Facilitation (PF)* PF is a structured approach to assist participating clinics with site-specific interventions focused on promoting workflow change [20]. Trained practice facilitators work with the clinic staff to identify each clinic’s incremental goals for change, developing a plan to accomplish the selected change, and evaluating outcomes and the need for modifications to the implemented processes. For this QI intervention, the project team developed materials pertinent to workflow optimization, including a summary of the health system’s opioid policy recommendations (Table 1), available EHR-based tools (e.g., “smartsets,” “smartphrases”), and general workflow recommendations for policy adherence. The PF portion of the intervention includes four elements: 1) Four to six PF sessions held over a 3–6 month period with clinic staff representing all clinical roles to identify opportunities and preferences for workflow improvements. 2) Encouraging the use of the Plan, Do, Study, Act (PDSA) model [21] to discuss and identify barriers, problem-solve, and summarize the implementation of actionable goals through small-scale tests of change in workflows. The identified changes are then implemented, and discussed in the subsequent PF session. 3) Identifying clinic-wide tools for effective communication between staff members. 4) Utilizing clinic-level outcome data to provide feedback on how the selected changes in workflow and clinical practices impact the clinic’s adherence to the opioid policy elements.
4. *Patient Education Materials* Two patient education videos, developed by a patient engagement and education organization, were made available for all clinics to provide to their patients: a five-minute video addressing treatment agreements, and a 20-min video focusing on opioid therapy in chronic pain [22]. Through the PF sessions, each clinic decided how to use the patient materials, such as making them a part of the pre-clinic visit or patient rooming process, having the patient watch them at home post-visit, or not use them at all.

**Table 2:** The intervention for augmenting routine health system-based implementation of opioid policy recommendations in primary care

QI Intervention Component	Description
Academic Detailing	A single on-site educational meeting between a content expert (project team member) and the clinicians and staff from the enrolled clinic wishing to improve the quality of care for their opioid-treated patients.
Two Online Educational Modules, delivered via email: 1) Responsible Opioid Prescribing 2) Shared Decision Making	Brief, straightforward, and easily accessible educational tools delivered via the web or mobile devices. A set of 20–21 multiple-choice questions with instant feedback allows learners to assess and validate their current knowledge of the targeted content, which is presented in the context of a given health system setting. These modules were developed by the project team members, content area experts, and reviewed by the health system and external experts (content can be made available upon request).
Practice Facilitation	An evidence-based method of assisting clinical practices in changing and optimizing the process of care. External facilitators (project team members) assist practices in implementing their prioritized goals and changing practice workflow, typically using the Plan, Do, Study, Act cycle model, ([21]) with the ultimate goal of improved patient care and outcomes.
Two Patient Education Modules: 1) Opioids for Chronic Pain 2) Agreement for Using Opioids	Brief, online educational tools for patients, professionally developed by Emmi Solutions, LLC (https://www.my-emmi.com/SelfReg/PAIN).

Participating clinicians and staff who complete all intervention components will receive 23 educational credits (American Medical Association Physician Recognition Award Category 1); for those completing a part of the intervention, the available credits will be prorated according to the documented participation.

#### Outcome measures

To evaluate the impact of the QI intervention, the project team will collect two main types of data before, during and after the intervention: a) EHR-based clinic-level data on elements of the health system’s opioid policy; and b) process measures from the clinical staff, and project team experiences, and perceptions related to the QI intervention implementation.

##### EHR-based Measures

(Table 3) The health system’s opioid policy contains numerous recommendations for optimizing care for patients with opioid-treated chronic pain (Table 1). Although the policy did not comment on opioid and benzodiazepine co-prescribing, we also chose to address this issue and track these data because of national guideline recommendations against the combination of such medications due to increased overdose risk [14, 15]. Aggregate clinic-level data will be collected monthly on the EHR-based measures that are both clinically important and reliably measured over time. Clinical adherence to only a handful of recommendations related to opioid prescribing practices can be reliably measured using the EHR data. Consistent with the health system’s opioid policy recommendations, the change in the clinic-level percentage of signed treatment agreements will serve as the primary outcome. While individual patient data on dispensed controlled substances are not available for outcome evaluation through the state PDMP database, we will measure the clinic-level rate of clinician/delegates signing into the PDMP, based on documentation of the PDMP check in the EHR. We will also assess selected EHR-based data on clinic- and clinician-level characteristics as covariates (e.g., FM/GIM, community/residency clinics, and patient panel size).

**Table 3:** Measures to evaluate the implementation of guideline and health system’s opioid management policy recommendations

Evaluation Component	Clinic-Level Measures
Clinically-Relevant Outcomes	
EHR-based Measures (aggregate clinic-level data)	
Treatment Agreement	Percent of eligible patients^a^ with signed treatment agreement in the past 12 months.
Urine Drug Testing	Percent of eligible patients^a^ with the health system-recommended urine drug testing completed in the past 12 months.
Opioid Therapy Risk Assessment	Percent of eligible patients^a^ with documented screening using the health system-recommended D.I.R.E. opioid misuse risk tool.
Depression Screening	Percent of eligible patients^a^ with documented screening using the health system-recommended PHQ-2 or − 9 depression screening tool.
Co-prescription of Opioids and Benzodiazepines^b^	Percent of eligible patients^a^ with presence of active prescriptions for both opioids and benzodiazepines.
PDMP Check	Percent of eligible patients^a^ with documented PDMP database check in the past 12 months.
Process Measures (aggregate clinic-level data)	
Clinic Team Surveys	Pre- and post-participation surveys will elicit: 1) ordinal responses as well as semi-qualitative comments to questions about current practice patterns; 2) comfort level with selected aspects of care for patients with opioid-treated chronic pain; 3) usefulness of the QI intervention components (post-participation).
Clinic Team Member Participation in the Intervention Components	Percent of clinicians and clinical staff per clinic who: - participated in the academic detailing session - enrolled in and completed each of the two online educational modules - participated in the practice facilitation sessions
Data from Practice Facilitators	Practice facilitator notes and experiences will enable identification of themes relevant to the implementation of the opioid policy (barriers and facilitators).

*D.I.R.E* Diagnosis, Intractability, Risk, Efficacy assessment tool, *QI* Quality Improvement, *PDMP* Prescription Drug Monitoring Program, *PHQ* Patient Health Questionnaire

^a^Target population: health system’s primary care adult (18 years old or older) patients treated with long-term opioids for chronic non-cancer pain. To be included in the analysis, patients must have met the following criteria: age ≥ 18 years old; active patient status (seen in the past 3 years) in the health system’s January 2016 panel data; have a primary care provider at the health system’s general internal medicine or family medicine clinics; do not have a diagnosis of malignant neoplasm (except non-melanoma skin cancer) or hospice status; and meet at least one of the two health system’s “opioid registry” criteria: Criterion 1: have at least one opioid prescription issued in the prior 45 days AND at least three opioid prescriptions issued in the prior 4 months; Criterion 2: have at least one opioid prescription issued in the prior 45 days, AND chronic pain diagnosis listed, AND a controlled substance agreement

^b^This element, although included in the opioid prescribing guidelines, was not a part of the health system’s policy on opioid therapy management

##### Process Measures

(Table 3) Clinician and clinic staff engagement and experience will be assessed through: A) quantitative and qualitative answers to pre- and post-participation questionnaires, developed by the project team (attached as an Additional file 1); B) prescriber and clinical staff participation in the QI intervention components (session attendance; enrollment in and completion of the online educational modules); and C) qualitative assessment of the experiences and perspectives of practice facilitators. In addition, we will also explore the number of log-ins into the online patient education tools.

### Statistical analysis

Sample size and power calculations were based on the health system’s EHR data from when the project was first planned in 2014 and on a cluster randomized trial methodology, with an intra-class correlation coefficient of 1.5%. These calculations estimated the project would have 84% power and over 95% confidence to detect a 20% relative increase in use of treatment agreements (primary outcome) over time [23]. A 20% increase is consistent with expert recommendations for measurement of a minimal clinically important difference [24]; given the short timeline of our intervention, even a minimal difference could suggest a meaningful change.

Longitudinal changes in clinic-level EHR-based measures will be assessed in the enrolled 9 clinics, as well as in the remaining 19 primary care clinics not enrolled in the QI project. The analysis of within- and between-clinic changes and experience with the intervention (process measures) will enable an evaluation of the intervention’s effects.

## Discussion

This paper describes the development of a multi-pronged, tailored QI intervention aimed at augmenting the system-wide implementation of policy and guidelines on opioid therapy management in chronic non-cancer pain. We hypothesize that the addition of this intervention will enhance implementation of guideline-driven recommendations in primary care, as compared to a “routine,” unenhanced policy rollout in a large health system. Rigorous evaluation of the effects of this intervention will be reported in a future publication.

Lack of efficient translation of research findings into routine practice is a common obstacle to improving the quality of care [11]. This may be particularly true for complex recommendations, such as those on opioid therapy management. Underutilization of opioid guidelines has been documented [3] and supported by our data on the baseline adherence of primary care clinicians to selected recommendations. Closing the gap between knowledge (guidelines) and practice can improve patient care and outcomes, which, in this case, could lead to curtailing the impact of opioid use disorders and overdose deaths [5–8].

It is not currently clear which methods are most effective for promoting system-level learning, change and QI in routine clinical care. A systematic and rigorous outcome assessment of QI efforts — such as those this project team proposes — is essential for discerning whether interventions with intuitive appeal actually result in desired change [25]. If the proposed enhanced efforts do not produce better outcomes than “routine” efforts, the health system is justified in not investing in such activities. If, on the other hand, these augmented efforts improve outcomes, the health system will be alerted to the fact that further investment in such implementation, although more labor-intensive, has clinical value. Therefore, positive or negative results should yield valuable information, promote system learning and change, and lay the foundation to improve approaches to future system-wide QI efforts.

### Conclusions

Developing methods for a health system-tailored QI intervention required a multi-step process to incorporate end-user feedback and account for the needs of targeted clinic team members. Delivery of such tailored QI interventions has the potential to enhance uptake of opioid therapy management policies in primary care. Results from this study are anticipated to elucidate the relative value of such QI activities.

## Additional file

Additional file 1 Surveys of the Clinic Team Members: A. Pre-Participation; B. Post-Participation. (DOCX 57 kb)

## Abbreviations

BPI: Brief Pain Inventory
CAM: Complementary and Alternative Medicine
D.I.R.E.: Diagnosis, Intractability, Risk, Efficacy assessment tool
EHR: Electronic Health Record
FM: family medicine
GIM: general internal medicine
MME: morphine milligram equivalent
PCP: Primary Care Provider
PDMP: Prescription Drug Monitoring Program
PDSA: Plan, Do, Study, Act model
PF: Practice Facilitation;
PHQ: Patient Health Questionnaire
QI: Quality Improvement
