# Supplementary material for: Enhancing system-wide implementation of opioid prescribing guidelines in primary care: protocol for a stepped-wedge quality improvement project
Source: BMC Health Serv Res. 2018 Jun 5;18:415. doi: 10.1186/s12913-018-3227-2 (PMC5989454; doi:10.1186/s12913-018-3227-2)
Supplement: Supplementary file 1 — Surveys of the Clinic Team Members: A. Pre-Participation; B. Post-Participation. (DOCX 57 kb) [file 12913_2018_3227_MOESM1_ESM.docx]

Additional File:

Surveys of the Clinic Team Members: A. Pre-Participation; B. Post-Participation.

A. Pre-Intervention Clinician and Staff Evaluation Site:________________

Date:_______________

**Improving Opioid Prescribing Practices & Patient Care**

Please answer these questions to help us evaluate the care of patients with opioid treated chronic pain in your clinic.

Please place an ‘X’ in the box for how much you agree or disagree with the following statements regarding opioid treated chronic pain patients. Opioid treated chronic pain patients are defined as adult patients who have been or are planned to be treated with opioids for greater than or equal to 3 consecutive months for chronic noncancer pain:

|  | *Strongly Disagree* | *Disagree* | *Neutral* | *Agree* | *Strongly*  *Agree* |
| --- | --- | --- | --- | --- | --- |
| 1. I am confident in my management of these patients. |  |  |  |  |  |
| 1. I want to learn more about the management of these patients. |  |  |  |  |  |
| 1. I plan to change how I manage these patients. |  |  |  |  |  |

Please place an ‘X’ in the box for the statement that most accurately reflects how often patients with opioid treated chronic pain are managed with the following:

|  | *Never* | *Rarely* | *Sometimes* | *Often* | *Very Often* |
| --- | --- | --- | --- | --- | --- |
| 4. Use of Patient Treatment Agreements |  |  |  |  |  |
| 5. Systematic screening for depression |  |  |  |  |  |
| 6. Routine urine drug screening |  |  |  |  |  |
| 7. Pain assessment with BPI (or a similar pain assessment tool) |  |  |  |  |  |
| 8. Assessment for risk of aberrant drug use behavior or addiction using D.I.R.E. (or a similar risk assessment tool) |  |  |  |  |  |
| 9. Functional assessment with BPI or FAQ5 |  |  |  |  |  |
| 10. Routinely check the Wisconsin PDMP |  |  |  |  |  |
| 11. Apply shared decision making |  |  |  |  |  |
| 12. Care team works together to manage these patients |  |  |  |  |  |

**
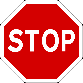
 Please STOP here, we will do the rest at the end of the presentation.**

Consider your commitment to change in your practice as a result of participating in this program: How likely are you to make changes in your current management of patients with opioid treated chronic pain?

| **Practice Changes** | *Very Unlikely* | *Unlikely* | *Neither*  *Likely or Unlikely* | *Somewhat Likely* | *Very Likely* |
| --- | --- | --- | --- | --- | --- |
| 13. Use of Patient Treatment Agreements |  |  |  |  |  |
| 14. Systematic screening for depression |  |  |  |  |  |
| 15. Routine urine drug screening |  |  |  |  |  |
| 16. Pain assessment with BPI or similar pain assessment tool |  |  |  |  |  |
| 17. Assessment for risk of aberrant drug use behavior or addiction using D.I.R.E. (or a similar risk assessment tool) |  |  |  |  |  |
| 18. Functional assessment with BPI or FAQ5 |  |  |  |  |  |
| 19. Routinely check the Wisconsin PDMP |  |  |  |  |  |
| 20. Apply shared decision making |  |  |  |  |  |
| 21. Care team works together to manage these patients |  |  |  |  |  |
| 22. Other changes? |  |  |  |  |  |
| 23. Describe these other changes: | | | | | |

| 24. What barriers do you anticipate as you change the way your practice manages patients with opioid treated chronic pain? |
| --- |

Please evaluate today’s academic detailing presentation.

|  | *Yes* | *No* |
| --- | --- | --- |
| 25. Presentation was effective |  |  |
| 26. Presentation was appropriate for my practice |  |  |
| 27. Information fair, balanced and free of commercial bias, if no explain: |  |  |

B. Post-intervention Clinician and Staff Evaluation Site:________________

Date:_______________

Improving Opioid Prescribing Practices & Patient Care

Please answer these questions to help us evaluate the care of patients with opioid treated chronic pain in your clinic.

Please place an ‘X’ in the box for how much you agree or disagree with the following statements regarding your current management of patients with opioid-treated chronic pain.

|  | *Strongly Disagree* | *Disagree* | *Neutral* | *Agree* | *Strongly*  *Agree* |
| --- | --- | --- | --- | --- | --- |
| I am confident in my management of these patients. |  |  |  |  |  |
| I want to learn more about the management of these patients. |  |  |  |  |  |
| I plan to change how I manage these patients. |  |  |  |  |  |

Please place an ‘X’ in the box for the statement that most accurately reflects how often you currently do the following in the management of patients with opioid-treated chronic pain:

|  | *Never* | *Rarely* | *Sometimes* | *Very Often* | *Extremely Often* | *Not a part of my role* |
| --- | --- | --- | --- | --- | --- | --- |
| Use of Treatment Agreements |  |  |  |  |  |  |
| Screen for depression |  |  |  |  |  |  |
| Complete urine drug screening |  |  |  |  |  |  |
| Assess pain using BPI or similar tool |  |  |  |  |  |  |
| Assess for risk of aberrant drug use behavior or addiction using D.I.R.E. (or similar risk assessment tool) |  |  |  |  |  |  |
| Assess function with BPI, FAQ5 or similar tool |  |  |  |  |  |  |
| Routinely check the Wisconsin PDMP database |  |  |  |  |  |  |
| Apply shared decision making |  |  |  |  |  |  |
| Work together with my care team to manage these patients |  |  |  |  |  |  |

Please place an ‘X’ in the box for how accurately each statement reflects how much positive change you made in your practice in the treatment of patients with opioid treated chronic pain in the past 3 months.

|  | *No Change* | *A Little Change* | *Some Change* | *Moderate Change* | *A Great Deal of Change* | *I already did this consistently* | *Not a part of my role* |
| --- | --- | --- | --- | --- | --- | --- | --- |
| Use of Treatment Agreements |  |  |  |  |  |  |  |
| Screen for depression |  |  |  |  |  |  |  |
| Complete urine drug screening |  |  |  |  |  |  |  |
| Assess pain using BPI or similar tool |  |  |  |  |  |  |  |
| Assess for risk of aberrant drug use behavior or addiction using D.I.R.E. (or similar risk assessment tool) |  |  |  |  |  |  |  |
| Assess function with BPI, FAQ5 or similar tool |  |  |  |  |  |  |  |
| Routinely check the Wisconsin PDMP database |  |  |  |  |  |  |  |
| Apply shared decision making |  |  |  |  |  |  |  |
| Work together with my care team to manage these patients |  |  |  |  |  |  |  |
| Other changes: |  |  |  |  |  |  |  |
| Describe other changes: | | | | | | | |

This initiative used a variety of educational activities and tools. Please place an ‘X’ in the box to reflect your view of their usefulness.

|  | *Not at all Useful* | *Slightly Useful* | *Somewhat Useful* | *Very Useful* | *Extremely Useful* | *Did Not Use/ Participate* |
| --- | --- | --- | --- | --- | --- | --- |
| Online education: Shared Decision Making module |  |  |  |  |  |  |
| Online education: Responsible Opioid Prescribing module |  |  |  |  |  |  |
| Academic Detailing Meeting |  |  |  |  |  |  |
| Practice Facilitation: 20 CME credit Improvement Project |  |  |  |  |  |  |
| Practice data |  |  |  |  |  |  |
| Participation in this program |  |  |  |  |  |  |

# Please rate the effectiveness of the practice facilitation sessions: Place an ‘X’ in the box for how much you agree or disagree with the following statements:

|  | *Strongly Disagree* | *Disagree* | *Neutral* | *Agree* | *Strongly*  *Agree* |
| --- | --- | --- | --- | --- | --- |
| Practice facilitation sessions addressed protocol and process changes needed to integrate safe opioid prescribing practices |  |  |  |  |  |
| Practice facilitation sessions provided ongoing support for shared learning at our clinic |  |  |  |  |  |
| Practice facilitation sessions provided tools and recommendations for the long-term sustainability of changes made |  |  |  |  |  |

| How could the practice facilitation sessions be improved? |
| --- |

In order to receive the 20 practice improvement CME credits, please complete these three reflection questions.

| **1. Based on changes you have made in your practice regarding the management patients with opioid treated chronic pain, what are the improvements in care that occurred?  If there were no improvements, do you have any thoughts as to why not?** |
| --- |

| **2. Reflect on the practice facilitation/quality improvement process itself. What have you learned from this process? For example, do you feel more confident in making changes in your practice, working with your team, or consulting practice facilitation staff?** |
| --- |

| **3. What part(s) of the practice facilitation/quality improvement process helped you the most for accomplishing change?** |
| --- |

**Title/Degree**: MD DO PA NP Other _______________________________________

**First Name**: _______________________________**Last Name:**____________________________________

**Email**: __________________________________________________________________________________

Abbreviations: BPI: Brief Pain Inventory; CME: Continuing Medical Education; D.I.R.E.: Diagnosis, Intractability, Risk, Efficacy assessment tool; FAQ5: Physical Functional Ability; PDMP: Prescription Drug Monitoring Program
